# Molecular diagnosis, genetic diversity and drug sensitivity patterns of *Mycobacterium tuberculosis* strains isolated from tuberculous meningitis patients at a tertiary care hospital in South India

**DOI:** 10.1371/journal.pone.0240257

**Published:** 2020-10-05

**Authors:** Krishnapriya Krishnakumariamma, Kalaiarasan Ellappan, Muthaiah Muthuraj, Kadhiravan Tamilarasu, Saka Vinod Kumar, Noyal Mariya Joseph

**Affiliations:** 1 Department of Microbiology, Jawaharlal Institute of Postgraduate Medical Education and Research (JIPMER), Pondicherry, India; 2 Intermediate Reference Laboratory, Government Hospital for Chest Diseases, Pondicherry, India; 3 Department of Medicine, Jawaharlal Institute of Postgraduate Medical Education and Research (JIPMER), Pondicherry, India; 4 Department of Pulmonary Medicine, Jawaharlal Institute of Postgraduate Medical Education and Research (JIPMER), Pondicherry, India; Institute for Integrative Biology of the Cell, FRANCE

## Abstract

Tuberculous meningitis (TBM) is the most severe form of *Mycobacterium tuberculosis* (Mtb) infection in humans and is a public health concern worldwide. We evaluated the performance of GeneXpert MTB/RIF (GeneXpert) for the diagnosis of TBM. In addition, genetic diversity and drug susceptibility profiling of Mtb strains isolated from TBM patients were also investigated. A total of 293 TBM suspected cerebrospinal fluid (CSF) samples were collected and subjected to GeneXpert and Mycobacterial Growth Indicator Tube (MGIT 960) culture, respectively. Sensitivity and specificity of GeneXpert was 72.7% and 98.5%, respectively by using MGIT 960 as a gold standard (GeneXpert (n = 20, 6.8%) vs MGIT 960 (n = 22, 7.5%)). All Mtb positive cultures were subjected to 24-locus Mycobacterial Interspersed Repetitive Unit Variable Number Tandem Repeat (MIRU-VNTR) typing, Line probe assay (LPA) and MGIT 960- Drug Susceptibility Testing (DST). The *rpoB* gene was amplified and sequenced for selected isolates. Among our TBM patients, East African Indian (EAI) lineage (n = 16, 72.7%) was most predominant followed by Beijing (n = 3, 13.6%), S-family (n = 2, 9.1%) and Delhi/CAS (n = 1, 4.5%). Three Mtb strains were found to be Isoniazid (INH) resistant by MGIT 960; however LPA revealed that two strains were INH resistant and one strain was multi drug resistant (MDR) (Resistant to Isoniazid and Rifampicin (RIF)). We identified rifampicin resistant isolate with the mutation D516F in rifampicin resistance-determining region (RRDR) and observed discordant results between LPA, GeneXpert and MGIT 960. In addition, GeneXpert showing false RIF resistance was identified (no mutation in RRDR). We conclude that GeneXpert is useful for the diagnostic confirmation of TBM; however a GeneXpert negative sample should be subjected to MGIT 960 culture or LPA to rule out TBM. EAI lineage was the most predominant among TBM patients in South India and associated with drug resistance. The discordance between GeneXpert, MGIT 960 and LPA with respect to rifampicin resistance has to be ruled out to avoid TB treatment failure or relapse.

## Introduction

Tuberculous meningitis (TBM) is the most severe form of extra pulmonary TB (EPTB) affecting the central nervous system (CNS) and it accounts for 1–5% of tuberculosis cases. TBM occurs when *Mycobacterium tuberculosis* (Mtb) released into the subarachnoid space, which could leads to the organization of inflammatory exudates in the basal cisterns that can produce obstruction to cerebrospinal fluid (CSF) flow, contributes to hydrocephalus and raised intracranial pressure [1]. Despite the accessibility of innovative health care, more than 50% of TBM patients may have neurological sequelae and the mortality ranged from 19.3% to 21.5% [2, 3]. The collective complications of multi drug resistance and HIV co-infection among TBM cases confer mortality close to 100% [4]. Hence, it is important for clinicians to diagnose TBM very early and choose the appropriate therapeutic options. Delay in TBM diagnosis may lead to poor outcomes and results in death. However, diagnosis of TBM remains challenge due to its pauci-bacillary nature of disease, especially in resource-constrained settings and TBM treatment is difficult due to the poor or incomplete penetration of anti-TB drugs across the blood–brain barrier and blood-CSF barriers. Generally, TBM diagnosis is based on a combination of clinical findings, various laboratory testing using CSF, and imaging findings. For TBM diagnosis, Ziehl–Neelsen (ZN) staining for Mtb is the most commonly available test, but without expert microscopists ZN staining has become insensitive and culture takes minimum two to three weeks to provide results with 30–60% sensitivity. The sensitivity of GeneXpert, a cartridge based rapid molecular test was still not high enough to rule out TBM (45–67% sensitivity to detect microbiologically proven TBM). GeneXpert Ultra has 70% sensitivity versus clinical case definition and misses up to 30% of cases [5]. However, a recent study from Donovan *et al*. reported that GeneXpert Ultra was not superior to GeneXpert for TBM diagnosis in both HIV-uninfected and HIV-infected adults [6]. This attracted our interest to validate the diagnostic performance of GeneXpert for the diagnosis of TBM by using Mycobacterial Growth Indicator Tube (MGIT 960) as a gold standard.

Mycobacterial interspersed repetitive units-variable number of tandem repeats (MIRU-VNTR) method have been used for studying the molecular epidemiology and transmission dynamics of Mtb. MIRU-VNTR defines the size and repeated number of units in each locus by amplification of interspersed repetitive units of mycobacteria and helps to study the diversity and clonal expansion of particular strain or lineages. Several studies suggest that the infecting Mtb strains from different lineages vary in their ability to obtain drug resistance, disease severity and pathogenesis [7, 8]. However, the influence of genetic factors that regulate the association of Mtb lineages with different level of disease including TBM remains unknown. Studies on Mtb lineages and drug resistance among TBM patients will highlight the molecular epidemiology of circulating bacteria and support in establishing the active control schemes. Delhi/CAS-type is the most predominant lineage of Mtb in North India followed by East African Indian (EAI) and Beijing [9–11]. However, EAI was predominant in South India as compared to Central and North India [12].

Earlier and accurate diagnosis of TBM continues to be a serious challenge for physicians and the consequences of delaying treatment are severe in India and worldwide. Although India has highest absolute number of tuberculosis cases including EPTB, there is paucity of data on prevailing genotypes and drug resistance profile of Mtb strains isolated from TBM cases in South India. Based on this knowledge, in this context, we aimed (i) to validate the diagnostic performance of GeneXpert to detect Mtb strains in CSF samples by using MGIT 960 as gold standard (ii) to understand the genetic diversity of circulating Mtb strains isolated from TBM patients from South India and (iii) to profile drug sensitivity patterns among Mtb strains isolated from TBM patients by using both phenotypic and genotypic methods

## Methods

### Study samples and Mtb cultures

We did an observational study to compare the performance of GeneXpert for the diagnosis of TBM by using MGIT 960 as a gold standard. A total of 293 CSF samples from TBM suspected cases were collected from a tertiary care hospital (JIPMER) during the study period from August 2018 to February 2020. A CSF volume of 2 ml was used for mycobacterial tests; 1.5 ml was used for GeneXpert and 0.5 ml for MGIT 960 culture. In addition, twenty two non-duplicate Mtb strains isolated from CSF samples were subjected to Mtb strain typing, MGIT 960 DST-SIRE [streptomycin (1.0 μg/ml), isoniazid (0.1 μg/ml), rifampicin (1.0 μg/ml) and ethambutol (5.0 μg/ml)] and Line Probe Assay (LPA).

### GeneXpert

The CSF samples were subjected to GeneXpert assay (Cepheid, New Delhi, India) according to manufacturer’s instructions [13]. The 1.5 ml of CSF samples diluted with GeneXpert MTB/RIF sample reagent (1.5 ml) was vortexed for 30 seconds and incubated at room temperature for 10 minutes. The CSF-reagent mixture was vortexed again for 30 seconds and then incubated for 5 minutes. The 2 ml of CSF-reagent mixture was then transferred into the GeneXpert MTB/RIF cartridge and loaded into the GeneXpert equipment. After 2 hours, the results were reported as Mtb positive or negative, and RIF resistant or sensitive among those positives.

### MGIT 960 culture

In this study, 500 μl of CSF sample was inoculated and incubated in the MGIT 960 system (Becton Dickinson India Private Limited, Gurgaon, India) till the tubes were flagged positive by the machine or till it was reported negative (42 days). The sterility of MGIT 960 positive cultures was checked on blood agar plates. MPT64 Ag test was performed for confirmation of *Mycobacterium tuberculosis* complex (MTBC) isolates recovered in MGIT 960 as per the manufacturer’s instructions. All isolates were preserved at -80°C for further studies

### DNA extraction and multiplex PCR

The DNA extraction from MGIT 960 Mtb culture was carried out using QIAamp DNA mini Kit (QIAGEN India Private Limited, New Delhi, India) as per the instructions provided by the manufacturer. The multiplex PCR (MPCR) was carried out to identify MTBC by using specific pair of primers designed to amplify IS6110 (123bp), protein b antigen (419bp) and MPB64 (240bp) [14]. The positive (H37Rv) and negative (Nuclease-Free Water, Sigma) controls were run concurrently during each multiplex PCR (MPCR) assay. The sequence of primers used to amplify IS6110, protein b antigen and MPB64 are listed in the Table 1. DNA amplification was performed in thermocycler (Eppendorf India Private Limited, New Delhi, India) using the following parameters; initial denaturation at 94°C for 5 min, then 40 cycles of denaturation at 94°C for 1 min, annealing at 65°C for 1.5 min, extension at 72°C for 1.5 min and final extension at 72°C for 10 min. The amplified DNA products were analysed by 1.5% agarose gel electrophoresis, stained with ethidium bromide (1%) and examined under UV light.

**Table 1:** Multiplex PCR targets primers.

Primers	Sequence	Product	Reference
**IS6110F**	5'CTCGTCCAGCGCCGCTTCGG3'	123bp	[14]
**IS6110R**	5'CCTGCGAGCGTAGGCGT3'		
**MPb64 F**	5'TCCGCTGCCAGTCGTCTTCC3'	240bp	
**MPb64 R**	5'GTCCTCGCGAGTCTAGGCCA3'		
**PabF**	5'ACCACCGAGCGGTTCGCCTGA3'	419bp	
**PabR**	5'GATCTGCGGGTCGTCCCAGGT3'		

### MIRU-VNTR

The DNA extraction from MGIT 960 Mtb positive culture was carried out as per the manufacturer’s instructions using QIAamp DNA mini Kit. The extracted DNA from Mtb isolates were subjected to 24 locus MIRU-VNTR typing as described by Supply *et al*. [15]. For each isolate, individual PCRs using primer sets for each locus was run accordingly. The number of repeats for each locus was calculated by the amplicon size. The strain lineage was confirmed by using the MIRU-VNTRplus web resource [16].

### Drug susceptibility testing (MGIT 960 DST-SIRE)

The Mtb positive cultures were sub-cultured onto MGIT 960 for drug susceptibility testing. Drug susceptibility to first-line anti-TB drugs including streptomycin (1.0 μg/ml), isoniazid (0.1 μg/ml), rifampicin (1.0 μg/ml) and ethambutol (5.0 μg/ml) were tested using SIRE Kit (Becton Dickinson India Private Limited, Gurgaon, India) in MGIT 960 liquid medium as per the manufacturer instructions. The susceptibility testing pattern was interpreted manually as fully susceptible, mono-resistant, or multidrug resistant. H37Rv was used as a control strain.

### Line Probe Assay

The Line Probe Assay (LPA), based on strip technology was used to detect rifampicin (RIF) resistance due to mutations in *rpoB* (RNA polymerase B subunit), as well as isoniazid (INH) resistance due to mutations in *kat G* (catalase peroxidase) and *inhA* (inoyl coenzyme A reducatse) genes. The Mycobacterial DNA was extracted as per the manufacturer’s instructions using GenoLyse kit (Hain Lifescience, Nehren, Germany) from all MGIT 960 Mtb positive strains. The multiplex PCR was carried out for the detection of *rpoB*, *katG*, *inhA* genes using 35μl of primer nucleotide mix, 10 μl of Taq DNA polymerase-PCR buffer mix and 5 μl of supernatant in a final volume of 50 μl. The amplification was carried out using the following PCR parameters: Initial denaturation of 15 min at 95°C, followed by 10 cycles of 30 sec at 95°C and 2 min at 65°C, and 20 cycles of 25 sec at 95°C, 40 sec at 50°C and 40 sec at 70°C and the extension step of 8 min at 70°C (1 cycle). The automated GT Blot 48 device (Hain Lifescience, Nehren, Germany) was used for hybridization and the results were interpreted as per the manufacturer’s instructions.

### *rpoB* gene amplification and sequencing

To analyse the mutations associated with RIF resistance, the rifampicin resistance-determining region (RRDR) was sequenced after amplification of *rpoB* gene by conventional PCR by using the primers, rpoBF (5’CAGACGTTGATCAACATCCG3’) and rpoBR (5’TACGGCGTTTCGATGAAC3’) to generate 305 bp that contains 81bp hot-spot RRDR. The extracted DNA (QIAamp DNA mini Kit) from the selected drug resistant Mtb strains including a reference strain (H37Rv) was subjected to PCR. The amplification was carried out using the following PCR parameters: Initial denaturation of 5 min at 94°C, followed by 35 cycles of 1 min at 94°C (denaturation), 30 sec at 50°C (annealing), 2 min at 72°C (extension) and the final extension step of 10 min at 72°C. The amplified product was subjected to 1.8% agarose gel electrophoresis and sequencing (ABI3500 Genetic analyser). The mutational analysis was carried out using BioEdit software [17].

### Statistical analysis

The diagnosis of TBM cases, gene positivity and RIF or INH resistance were expressed as percentages. The sensitivity and specificity of GeneXpert was calculated using MGIT 960 culture as a gold standard. The SPSS software (Version 20) was used for the statistical analysis.

### Ethical statement

The study was reviewed and approved by the Institute Ethics Committee (IEC) (Human studies) at Jawaharlal Institute of Postgraduate Medical Education and Research (JIPMER), Pondicherry (Approval No. JIP/IEC/2019/143). The IEC granted waiver of consent for this study. In this study, we utilized only the samples routinely sent to the mycobacteriology laboratory for diagnosis of TBM. The patient samples are not used for other purposes besides diagnosis of TBM and characterization of the Mtb isolates.

## Results

### Characteristics of the study population

Of the total 293 patients <1year were 4 (1.4%), 1 to 14 years were 32 (10.9%), 15 to 30 years were 85 (29%), 31–60 years were 137 (46.8) and >60 years were 35 (11.9%). Of the 293 patients, 164 (56%) were male and 129 (44%) were female.

### Performance of GeneXpert and MGIT 960 culture on CSF samples

**Fig 1:**
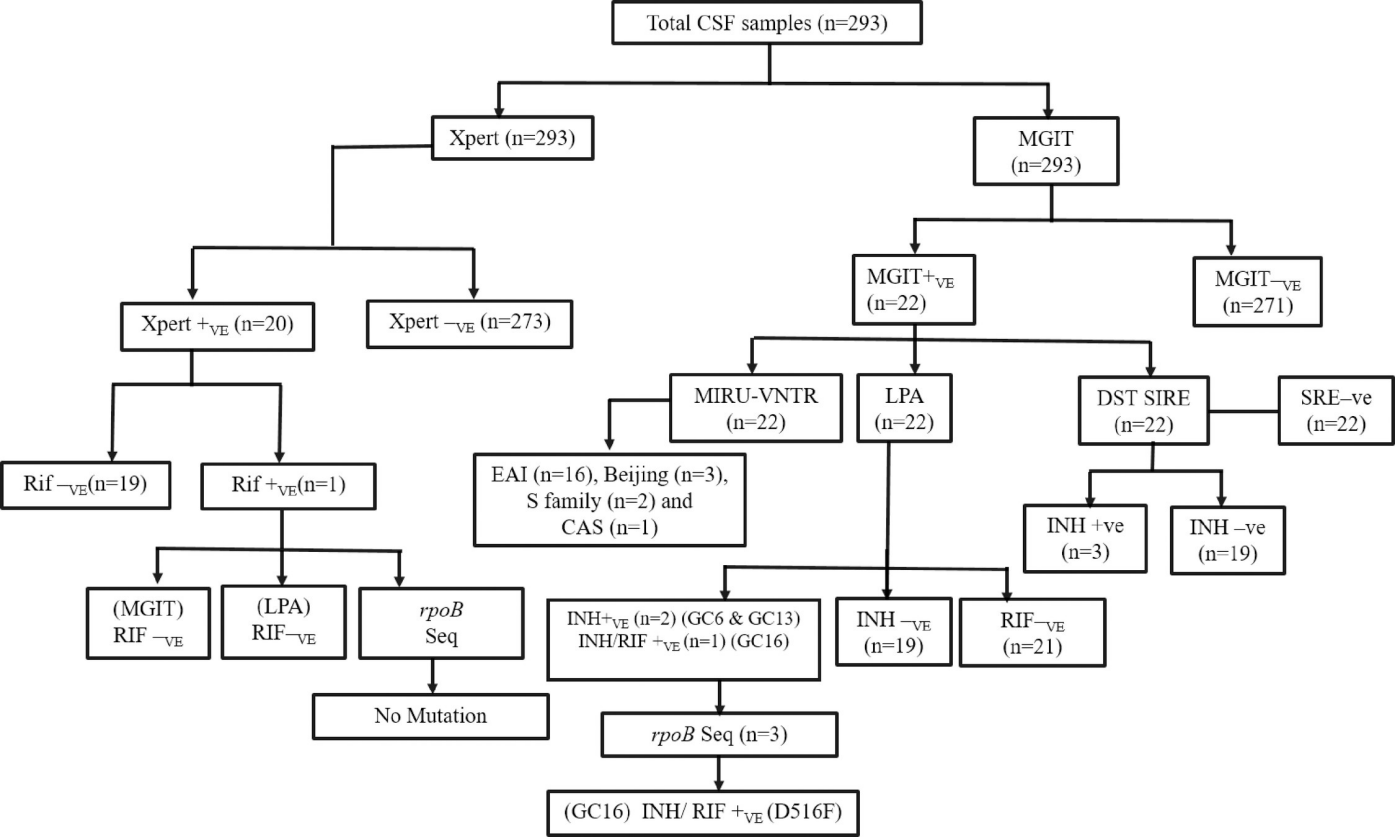
Diagnostic algorithm of the study. Xpert—GeneXpert; MGIT—MGIT 960; +ve—Positive;–ve—Negative; S—streptomycin; I—isoniazid; R—rifampicin and E—ethambutol.

Of the total 293 patients, 20 (6.8%) were GeneXpert positive and 22 (7.5%) MGIT 960 culture positive (Fig 1). All MGIT 960 cultures were found to be positive for MPT64 Ag Immunochromatographic test and Mtb species specific Multiplex PCR assay. Of the total 20 GeneXpert positive samples, 16 and 4 were MGIT 960 culture positive and negative, respectively. Among 22 MGIT positive cases, 16 and 6 were GeneXpert positive and negative, respectively (Table 2). The sensitivity and specificity of GeneXpert was 72.7% and 98.5%, respectively by using MGIT 960 culture as a gold standard.

**Table 2:** Comparison of GeneXpert assay by using MGIT 960 as the gold standard.

		MGIT 960 culture		Total
		Positive	Negative	
**GeneXpert**	**Positive**	16	4	20
	**Negative**	6	267	273
**Total**		22	271	293

### Phenotypic and genotypic drug susceptibility profiling

Out of 22 MGIT positive cultures, three strains (GC6, GC13 and GC16) were found to be INH resistant by MGIT-DST. Line Probe Assay (LPA) assay revealed that among 22 MGIT positive cultures two Mtb strains (GC6 and GC13) were INH resistant and one (GC16) was multi drug resistant (INH and RIF resistant) (S1 Table in S1 File). The *rpoB* gene sequencing revealed that GC16 strain harbour detectable mutation GAC516TTC (D516F) in 81bp RRDR (S1-S3 Figs in S1 File). However, this MDR (GC16) strain was not detected by GeneXpert in CSF sample. Among 20 GeneXpert positive cases, one was detected as RIF resistant but Mtb (GC6) isolated from this sample was found to be rifampicin sensitive by both MGIT 960 and LPA and no mutation was observed in RRDR. In addition, we observed that this GC6 Mtb strain was found to be INH resistant by both MGIT and LPA. Hence, our results revealed the discordance results between GeneXpert, MGIT 960 and LPA with respect to rifampicin resistance (S1 Table in S1 File). Importantly, we observed that the LPA had a better performance to GeneXpert and MGIT-DST with regards to the detection of RIF resistance; however, LPA and MGIT-DST showed similar drug susceptibility patterns with respect to INH resistant.

### MIRU-VNTR

In this tertiary care hospital from South India, the majority of Mtb strains were EAI lineage (n = 16, 72.7%) followed by Beijing (n = 3, 13.6%), S-family (n = 2, 9.1%) and Delhi/CAS (n = 1, 4.5%). The drug resistant strains, GC6 and GC16 belonged to EAI and G13 was Delhi/CAS lineage (S1 Table in S1 File).

## Discussion

The diagnosis of TBM remains a challenge for clinicians worldwide due to paucibacillary nature of the disease [18]. In 2011, World Health Organization (WHO) recommended GeneXpert over CSF smear and culture as the initial diagnostic tests for patients suspected to have TB or drug resistant TB and for patients suspected to be having TBM [19–21]. Several studies have reported that GeneXpert is a useful diagnostic test to rule out TBM cases from CSF samples [22, 23]. In addition, numerous studies also reported that GeneXpert ultra was superior to GeneXpert for the diagnosis of TBM [24–26]; however, a recent study by Donovan *et al*., reported that GeneXpert Ultra was not superior to GeneXpert for the diagnosis of TBM in HIV-uninfected and HIV-infected patients [6]. In our study, we compared the diagnostic performance of GeneXpert for detecting Mtb in CSF samples by using MGIT 960 as a gold standard. Our results showed that GeneXpert had 72.7% sensitivity and 98.5% specificity for diagnosing TBM by using MGIT 960 as gold standard. We observed that of the total 293 samples, 22 were positive for MGIT 960 culture and 20 were GeneXpert positive (Table 2). Importantly, of the 20 cases detected by GeneXpert, four were not detected by MGIT culture and one rifampicin resistant Mtb detected by GeneXpert was found to be sensitive by both LPA and MGIT DST-SIRE and no mutation in *rpoB* gene. It’s unlikely, the false positive (GeneXpert positive but culture-negative) may have occurred due to cross-contamination, as GeneXpert cartridges have closed chambers [27]. Another reason for false GeneXpert positive may be due to commencement of empirical treatment for TBM patients before the culture results were known. Of the 22 cases detected by MGIT 960, six were not detected by GeneXpert. False negative results in GeneXpert may be due to the presence of less bacillary load (<100 CFU/ml) in CSF samples [22] or the presence of PCR inhibitors in CSF samples that may inhibits PCR considerably and cause false-negative GeneXpert results [28]. Interestingly, studies also reported that centrifugation and suspension of CSF samples is likely to remove PCR inhibitors [29]. An alternative reason for false negative GeneXpert results may be due to inappropriate capture or lysis of all bacteria within the cartridge during the process [22]. As recommended by WHO, TBM suspected patient who receive a negative result by GeneXpert should undergo further diagnostic tests.

The association between Mtb lineages and disease severity, emergence of drug resistance, host response and transmissibility in TBM are highly scarce and alarming. In the present study, we investigated the genome diversity of 22 Mtb strains isolated from CSF samples using 24 MIRUs (the 24 loci panel). MIRU-VNTR typing is a molecular genotyping method that uses counts of the number of times pre-specified loci repeat in a strain. This study gives an insight into the genetic diversity of Mtb strains isolated from CSF samples in South India. The predominant genotype among our TBM cases in South India was that of EAI lineage (n = 16, 72.7%) followed by Beijing (n = 3, 13.6%), S-family (n = 2, 9.1%) and Delhi/CAS (n = 1, 4.5%). A study from Varghese *et al*. reported that EAI displayed significant association to CNS TB (p-0.04) [30]. The EAI lineage strains are the most ancient Mtb strain found mostly in the southern part of India, particularly prevalent in tropical countries. EAI lineage contributes to 84% of Mtb isolates in South India [31]. A study from North India reported that EAI is the second most frequently isolated lineage (22.8%) from CSF samples [12]. Our study results depict an extreme geographical difference between South India and North India. In northern part of India, Delhi/CAS is predominant strain whereas in western India, the dominant strain is Beijing strain. Hence, understanding the genetic diversity of Mtb strains in a given clinical setting may play an important role to introduce more effective control measures and management strategies against PTB and EPTB including TBM.

Drug susceptibility profiles of Mtb strains isolated from CSF samples were analysed by phenotypic (MGIT-DST SIRE) and genotypic (GeneXpert, Line Probe Assay (LPA) and *rpoB* sequencing) methods. Our results highlighted that LPA had a better performance compared to GeneXpert and MGIT 960 with regards to the detection of RIF resistance. Of the total of 22 Mtb strains, three were INH resistant by both MGIT 960 and LPA. A CSF sample which was detected as RIF resistant by GeneXpert was found to be RIF sensitive by both MGIT 960 and LPA and no mutation was observed in RRDR of *rpoB* gene. Importantly, a GeneXpert negative CSF sample which was cultured in MGIT 960 was found to be INH and RIF resistant by LPA and mutation (GAC516TTC, D516F) was observed in RRDR. Interestingly, this isolate was RIF sensitive by MGIT-DST SIRE. Although, the association of D516F substitution with discordant susceptibility tests was reported in previous studies [32, 33], our data was not in agreement with the results of Tan *et al*., Berrada *et al*., and Aragon *et al*. [34–36]. The occurrence of mutation D516F has been proposed to cause low-level rifampicin resistance [32, 37]. Low level rifampicin resistance may influence the treatment; particularly phenotypic DST outcomes may vary from the actual effectiveness of anti-TB drugs in patients [38]. Although MDR Mtb strains are resistant to at least rifampicin and isoniazid, the key factor for treatment failure is rifampicin resistance. GeneXpert and LPA are the two WHO approved molecular diagnostic tests that detect mutations in the RRDR of the *rpoB* gene for the earlier diagnosis of rifampicin resistance in clinical specimens and culture isolates. However, these tests are not specific and they do not detect mutations outside of the 81bp RRDR of the *rpoB* gene of Mtb, which contains 95% of known rifampicin resistance-associated codons, leading to false-negative rifampicin resistance [13, 39, 40]. In addition, MGIT 960 culture, which is the current standard resistance test endorsed by the WHO to validate novel molecular assays may miss occult rifampicin resistance due to ‘disputed’ mutations [39]. This is due to the pre-set standard conditions of rifampicin critical concentration of 1 μg/ml. Hence, our results highlighted that the discordance between GeneXpert MTB/RIF, LPA and MGIT 960 culture may cause laboratory error in detecting rifampicin resistance in TBM, which leads to treatment failure or relapse. In addition, we highlighted that the drug resistant strains, GC6 and GC16 belonged to EAI and G13 was Delhi/CAS lineage.

In summary, although this study was limited by a relatively small sample size our study highlighted the diagnostic usefulness of GeneXpert for TBM. GeneXpert negative results do not rule out TBM and we strongly recommend that the CSF sample which was found to be negative by GeneXpert should be subjected to culture or LPA or other diagnostic assays. Genetic diversity of Mtb strains isolated from TBM patients from South India was highlighted; among different genotypes EAI was the most predominant and was associated with drug resistance among TBM patients followed by Delhi/CAS. In addition, our results revealed the discordance between GeneXpert MTB/RIF, LPA and MGIT 960 culture with respect to rifampicin resistance which may lead to treatment failure or relapse. Hence we suggest that further studies on these subjects may improve the diagnostic performance and patient management for TBM.

## Supporting information

S1 File (DOCX)
