# Supplementary material for: Molecular diagnosis, genetic diversity and drug sensitivity patterns of Mycobacterium tuberculosis strains isolated from tuberculous meningitis patients at a tertiary care hospital in South India
Source: PLoS One. 2020 Oct 5;15(10):e0240257. doi: 10.1371/journal.pone.0240257 (PMC7535050; doi:10.1371/journal.pone.0240257)
Supplement: S1 File — (DOCX) [file pone.0240257.s001.docx]

**Molecular diagnosis, genetic diversity and drug sensitivity patterns of *Mycobacterium tuberculosis* strains isolated from tuberculous meningitis patients at a tertiary care hospital in South India**

Krishnapriya Krishnakumariamma^1¶^, Kalaiarasan Ellappan^1¶^, Muthaiah Muthuraj^2^, Kadhiravan Tamilarasu^3^, Saka Vinod Kumar^4^, Noyal Mariya Joseph^1*^

^1^Department of Microbiology, Jawaharlal Institute of Postgraduate Medical Education and Research (JIPMER), Pondicherry, India

^2^Intermediate Reference Laboratory, Government Hospital for Chest Diseases, Pondicherry, India

^3^Department of Medicine, Jawaharlal Institute of Postgraduate Medical Education and Research (JIPMER), Pondicherry, India

^4^Department of Pulmonary Medicine, Jawaharlal Institute of Postgraduate Medical Education and Research (JIPMER), Pondicherry, India

^¶^ These authors contributed equally to this work.

***Corresponding author**

E-mail: [noyaljoseph@yahoo.com](mailto:noyaljoseph@yahoo.com) **(NMJ)**

**Table of Contents**

S1 Table**.** Detailed results obtained including demographic, drug resistance, diagnostic performance of GeneXpert for respective CSF samples and 24 loci MIRU-VNTR from 22 Mtb strains isolated from CSF samples in south India………………………………Page 3

S1 Fig. Amplification of *rpoB* gene (305bp) from H37Rv, GC6 and GC16 strains. L1: 100bp ladder, L2: H37Rv, L3: GC6 (Xpert RIF resistant, MGIT RIF sensitive, LPA RIF sensitive and no mutation in RRDR), L4: GC16 (Xpert Mtb not detected, MGIT RIF sensitive, LPA RIF resistant and mutation in RRDR(D516F))…………………………………………………………………………………………..Page 4

S2 Fig. Electropherogram of amplified RRDR sequence ……………………………………………………………………..……………..Page 5

S3 Fig. Mutational analysis in RRDR sequence …………………………………………………………………………………..……………Page 6

**S1 Table.** **Detailed results obtained including demographic, drug resistance, diagnostic performance of GeneXpert for respective CSF samples and 24 loci MIRU-VNTR from 22 Mtb strains isolated from CSF samples in south India**

| **Patients**  **(MGIT culture)** | **Year** | **Sex** | **Age** | **MIRU-VNTR profile** | **Lineage** | **Distance** | **MGIT960 culture DST** | | | | **LPA** | | **GeneXpert from CSF samples** | |
| --- | --- | --- | --- | --- | --- | --- | --- | --- | --- | --- | --- | --- | --- | --- |
|  |  |  |  | ETR-A, ETR-B, ETR-C, ETR-D, ETR-E, MIRU 2, MIRU 10, MIRU 16, MIRU 20, MIRU 23, MIRU 24, MIRU 26, MIRU 27, MIRU 39, MIRU 40, Mtub04, Mtub21, QUB-11b, Mtub29, Mtub30, Mtub34, Mtub39, QUB-26, QUB-4156 |  |  | S | I | R | E | INH | RIF | Mtb status | RIF |
| GC1 | 2018 | F | 51 | 8 4 2 6 5 2 4 3 2 6 2 2 3 1 3 - 10 3 4 2 3 9 4 2 | EAI | 0.34 | s | s | s | s | s | s | Mtb detected | s |
| GC2 | 2018 | M | 24 | 6 1 4 7 4 2 5 4 2 6 2 3 3 3 4 1 6 3 3 1 3 4 4 1 | EAI | 0.29 | s | s | s | s | s | s | Mtb detected | s |
| GC3 | 2018 | M | 55 | 6 1 4 6 5 2 5 3 2 6 2 2 3 4 4 3 7 3 3 1 3 4 5 2 | EAI | 0.33 | s | s | s | s | s | s | Mtb detected | s |
| GC4 | 2018 | M | 38 | 2 2 4 3 3 2 3 1 2 7 1 5 3 2 4 3 2 4 4 2 2 5 5 2 | S family | 0.29 | s | s | s | s | s | s | Mtb detected | s |
| GC5 | 2018 | F | 49 | 6 4 4 10 6 3 5 4 3 6 3 2 3 4 2 6 6 4 3 1 3 4 7 2 | EAI | 0.54 | s | s | s | s | s | s | Mtb detected | s |
| GC6 | 2018 | F | 25 | 6 4 4 6 5 2 5 4 2 7 2 2 3 2 2 2 6 3 3 1 2 4 6 1 | EAI | 0.33 | s | r | s | s | r | s | Mtb detected | r |
| GC7 | 2018 | F | 30 | 6 – 4 6 – 2 5 3 – 6 2 2 3 4 3 1 3 – 2 1 3 3 8 1 | EAI | 0.35 | s | s | s | s | s | s | Mtb detected | s |
| GC8 | 2018 | M | 21 | 2 2 4 3 3 2 – 1 2 7 1 5 3 2 4 2 2 4 4 2 2 5 - 2 | S family | 0.31 | s | s | s | s | s | s | Mtb not detected | - |
| GC9 | 2018 | M | 26 | 9 5 2 6 5 2 4 3 2 6 2 2 3 1 3 3 10 3 3 2 3 5 4 1 | EAI | 0.33 | s | s | s | s | s | s | Mtb not detected | - |
| GC11 | 2018 | F | 28 | 5 6 4 3 5 2 4 3 3 6 2 2 3 3 3 2 4 5 3 2 3 4 6 2 | EAI | 0.29 | s | s | s | s | s | s | Mtb detected | s |
| GC12 | 2018 | F | 40 | 3 2 – 3 6 3 3 3 3 5 1 7 3 3 3 4 5 4 4 4 3 4 8 2 | BEIJING | 0.34 | s | s | s | s | s | s | Mtb detected | s |
| GC13 | 2019 | M | 65 | 4 2 2 3 5 2 5 5 2 5 1 3 3 3 3 5 4 2 4 2 3 3 8 5 | DELHI/CAS | 0.25 | s | r | s | s | r | s | Mtb detected | s |
| GC14 | 2019 | F | 46 | 6 1 4 6 4 2 4 3 2 5 2 2 3 2 3 1 6 3 3 1 3 4 6 1 | EAI | 0.25 | s | s | s | s | s | s | Mtb not detected | - |
| GC15 | 2019 | F | 30 | 6 1 4 6 4 2 5 4 3 – 3 2 3 4 4 3 6 3 3 2 3 4 5 1 | EAI | 0.34 | s | s | s | s | s | s | Mtb detected | s |
| GC16 | 2019 | F | 25 | 7 3 4 – 4 2 6 5 – 7 3 2 3 4 3 3 6 10 4 2 3 4 6 1 | EAI | 0.54 | s | r | s | s | r | r | Mtb not detected | - |
| GC17 | 2019 | M | 44 | 6 1 4 6 4 1 4 3 2 7 2 2 3 3 4 2 6 3 3 1 – 4 4 1 | EAI | 0.17 | s | s | s | s | s | s | Mtb detected | s |
| GC18 | 2019 | M | 45 | 4 2 4 3 5 2 2 2 2 5 1 6 3 3 1 4 7 5 4 3 – 3 8 2 | BEIJING | 0.34 | s | s | s | s | s | s | Mtb detected | s |
| GC19 | 2019 | M | 64 | 4 4 4 6 6 2 4 3 2 10 3 2 3 3 3 2 10 3 3 1 3 6 7 2 | EAI | 0.37 | s | s | s | s | s | s | Mtb detected | s |
| GC20 | 2019 | M | 53 | 6 1 4 5 4 2 4 4 2 6 2 2 4 4 4 2 4 3 3 1 3 4 4 2 | EAI | 0.29 | s | s | s | s | s | s | Mtb not detected | - |
| GC22 | 2019 | F | 26 | 4 3 4 3 5 2 – 2 2 4 1 5 4 4 3 3 3 5 4 4 3 3 8 2 | BEIJING | 0.34 | s | s | s | s | s | s | Mtb detected | s |
| GC23 | 2019 | F | 35 | 9 5 4 1 5 2 4 3 1 6 2 2 3 3 3 2 6 – 3 2 3 4 5 1 | EAI | 0.26 | s | s | s | s | s | s | Mtb detected | s |
| GC24 | 2019 | F | 62 | 6 1 4 6 4 2 5 4 2 6 2 – 3 4 5 2 6 3 3 1 3 2 6 1 | EAI | 0.26 | s | s | s | s | s | s | Mtb not detected | - |
| H37Rv | Lab Strain | | | 3 3 4 3 3 3 3 2 2 6 1 3 3 2 1 2 2 5 4 2 3 5 5 2 | H37rv | 0.08 | s | s | s | s | s | s | Mtb detected | s |


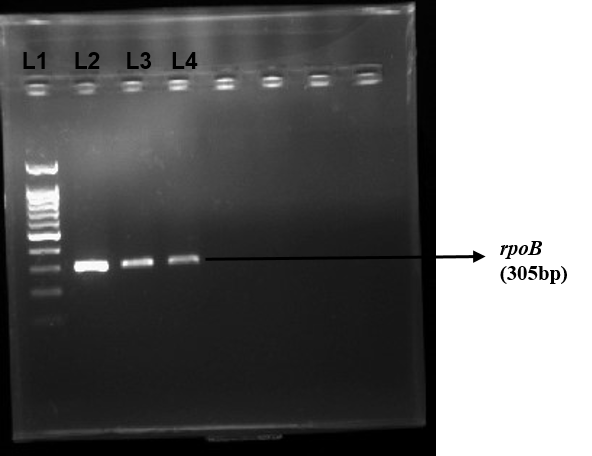


**S1 Fig. Amplification of *rpoB* gene (305bp) from H37Rv, GC6 and GC16 strains. L1: 100bp ladder, L2: H37Rv, L3: GC6 (Xpert RIF resistant, MGIT RIF sensitive, LPA RIF sensitive and no mutation in RRDR), L4: GC16 (Xpert Mtb not detected, MGIT RIF sensitive, LPA RIF resistant and mutation in RRDR(D516F)).**


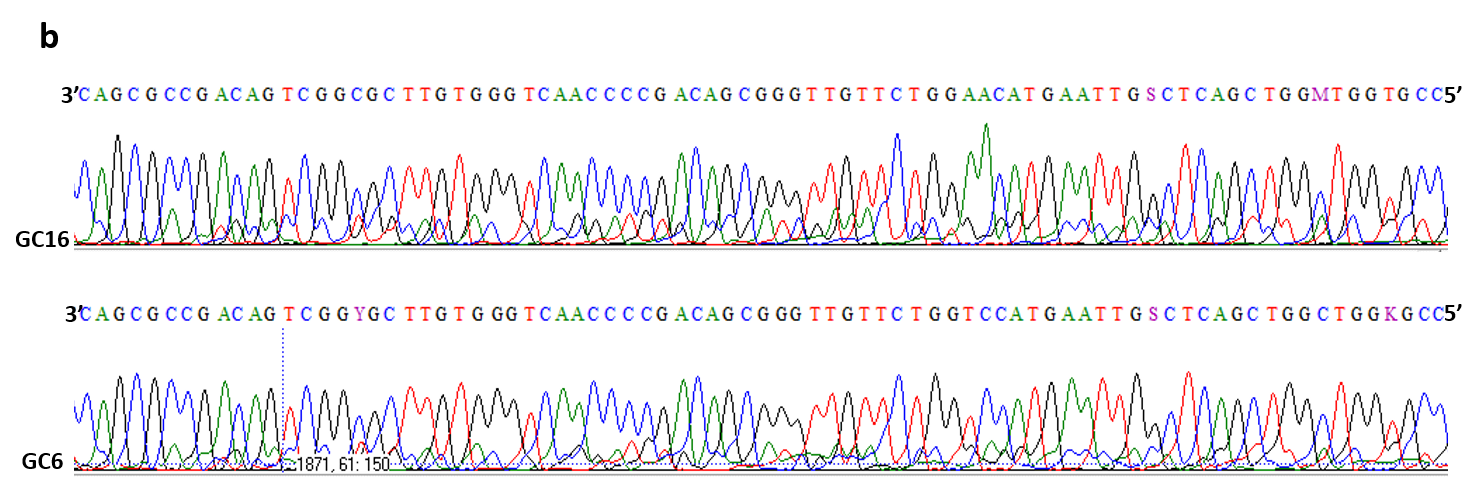


**S2 Fig. Electropherogram of amplified RRDR sequence**


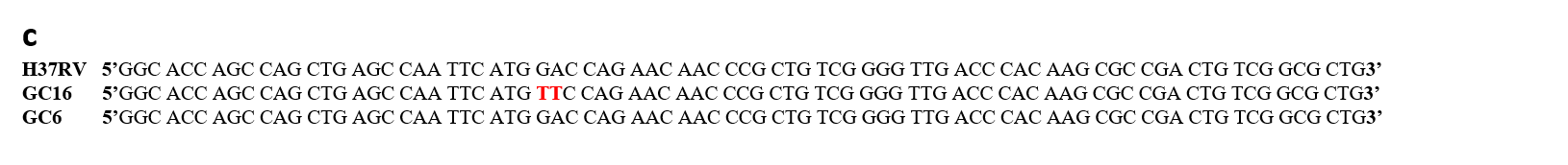


**S3 Fig. Mutational analysis in RRDR sequence**
