# Supplementary material for: Molecular diagnosis, genetic diversity and drug sensitivity patterns of Mycobacterium tuberculosis strains isolated from tuberculous meningitis patients at a tertiary care hospital in South India
Source: PLoS One. 2020 Oct 5;15(10):e0240257. doi: 10.1371/journal.pone.0240257 (PMC7535050; doi:10.1371/journal.pone.0240257)
Supplement: Attachment — Submitted filename: Response to reivewers 04-09-2020.docx [file pone.0240257.s002.docx]

**Authors Reply**

Reviewer #1:

**Thank you so much for reviewing our manuscript and your suggestions are very helpful. As per the reviewer’s suggestions, we revised the manuscript accordingly and highlighted the corrections.**

a) **MGIT 960 is presented in two ways: MGIT 960 and MGIT960**

We presented MGIT 960 as MGIT 960 throughout the manuscript

b) **In the method section it is stated that the 2 mL of the CSF were divided into 1.5 mL for Xpert and 0.5 mL for MGIT, then it says that 2 mL were used for Xpert**

We have written this protocol elaborately in GeneXpert methodology section. The 1.5 ml of CSF samples diluted with GeneXpert MTB/RIF sample reagent (1.5 ml) was vortexed for 30 seconds and incubated at room temperature for 10 minutes. The CSF-reagent mixture was vortexed again for 30 seconds and then incubated for 5 minutes. The 2 ml of CSF-reagent mixture was then transferred into the GeneXpert MTB/RIF cartridge and loaded into the GeneXpert equipment. Kindly find the revised sentence in the page no 4

c) **To standardize the wording of units in relation to their values, or with space or without space, observe the recommendations of PLOS**

As per the guidelines of PLOS, we provided the space to units in relation to their values. Kindly find the corrections in the page no’s 4, 5, 6 and 9

d) **Standardize the wording of the names of the drugs, or start with a capital letter or a small letter**

We started the names of all the drugs with a small letter. SIRE was written as streptomycin, isoniazid, rifampicin and ethambutol. Kindly find the changes in the page no’s 4 and 6

e) **Check if the parameters of the multiplex PCR for the rpoB, inhA and katG genes are correct**

As per the reviewer’s suggestion, we have checked the parameters of the multiplex PCR for the *rpoB, inhA* and *katG* genes and corrected appropriately. Please find the changes in page 6

f) **I recommend a careful review of the references, mainly related to how the species M. tuberculosis is being written;**

As per the reviewer’s suggestion, we revised the reference very carefully and italicised *Mycobacterium tuberculosis* throughout the reference section.

g) **The highlighted isolates (GC3, GC13 and GC16) throughout the text and in the legends are spelled in different ways**

Thank you so much for this comment. We corrected the isolates no’s as GC6, GC13 and GC16. Kindly find the changes in the supporting information in (S1-S3 Figs in S1File).

h) **The purpose of the presentation of Figure 2B is not clear, since the electropherograms do not correspond to the sequences presented for the *M. tuberculosis* H37Rv strain, and the clinical isolates GC6 and GC16**

To analyse the mutations associated with RIF resistance, the rifampicin resistance-determining region (RRDR) was sequenced after amplification of *rpoB* gene by conventional PCR by using the primers, rpoBF (5’CAGACGTTGATCAACATCCG3’) and rpoBR (5’TACGGCGTTTCGATGAAC3’) to generate 305 bp that contains 81bp hot-spot RRDR (Page No 6). The *rpoB* gene was sequenced using reverse primer and the electropherogram of RRDR sequence was presented as 3’ to 5’ (S2 Fig in S1 File). During mutation analysis, we have converted the sequence into 5’ to 3’ (S3 Fig in S1 File). We revised the figures accordingly. Kindly find the changes in the S1 File. We compared the *rpoB* sequence of GC6 and GC16 strains with H37Rv *rpoB* (KY702773.1)

i) **Figure 3 shows the scientific quality and accuracy of the results obtained in this study, however it does not contribute to a better understanding of the work, I recommend removing it**

As per the reviewer’s suggestion, we removed the figure 3 from the manuscript

j) **MIRU-VNTR is recognized as a fundamental tool for the molecular characterization of *M. tuberculosis* isolates and has been widely used for this purpose, therefore it is not necessary to include the table of primers used for PCR reactions, unless this work proposed some modifications in these sequences**

As per the reviewer’s suggestion, we removed the Primers list from the manuscript. However, we included the references in the MIRU-VNTR methodology section.

k) **Although it is not the objective of this work, I believe it is important to include in the discussion information that discards epidemiological links of the patients involved in this study**

Thank you so much for your comments. We aimed this study to validate the diagnostic performance of GeneXpert to diagnose TBM by using MGIT 960 as the gold standard, in addition to identify the genotypes and drug susceptibility profiling of circulating Mtb strains among TBM patients in south India. The discordant results between GeneXpert, MGIT 960 and Line probe assay with respect to rifampicin resistance was highlighted. Hence, we didn’t discuss the epidemiological link of the TBM patients. In the present study, we suggest that despite the presence of diverse genotypes of Mtb strains in south India, the EAI family is the most predominant Mtb genotype linked to drug resistant TB among south Indian TBM patients. We consider that the EAI family is driving drug resistant TB in south India and assume that its role in association with multi drug resistant TB will increase in the future. However, epidemiological surveillance of these cases in future may find new patients from their epidemiological links, predict risk factors and develop strategies to control and for the management of drug resistant TB in south India.

l) **In this manuscript the authors refer to: “confirmation of isolates of MBTC” and “identify of MBTC” however this acronym is not defined throughout the text, if this acronym refers to the M. tuberculosis Complex, consider the adequacy as the publication by Riojas et al., Int J Syst Evol Microbiol 2018; 68: 324–332.**

The acronym of *Mycobacterium tuberculosis* complex is MTBC. We made the changes in the manuscript and highlighted in the page 5.

**Reviewer #2: accept as is.**

Thank you so much for your valuable time and review
