# Supplementary material for: Molecular diagnosis, genetic diversity and drug sensitivity patterns of Mycobacterium tuberculosis strains isolated from tuberculous meningitis patients at a tertiary care hospital in South India
Source: PLoS One. 2020 Oct 5;15(10):e0240257. doi: 10.1371/journal.pone.0240257 (PMC7535050; doi:10.1371/journal.pone.0240257)
Supplement: Peer review history

# Peer review: Decision Letter 0

**DOI:** 10.1371/journal.pone.0240257.r001

**Authors:** Christophe Sola

25 Aug 2020

PONE-D-20-08897

Molecular diagnosis, genetic diversity and drug sensitivity patterns of Mycobacterium tuberculosis strains isolated from tuberculous meningitis patients at a tertiary care hospital in South India

PLOS ONE

Dear Dr. Joseph,

Thank you for submitting your manuscript to PLOS ONE. After careful consideration, we feel that it has merit but does not fully meet PLOS ONE’s publication criteria as it currently stands. Therefore, we invite you to submit a revised version of the manuscript that addresses the points raised during the review process.

==============================

ACADEMIC EDITOR: please confer to the minor modifications suggested by reviewer 1.. Reviewer 2 did accept the manuscript without changes.

==============================

Please submit your revised manuscript by Oct 09 2020 11:59PM. If you will need more time than this to complete your revisions, please reply to this message or contact the journal office at plosone@plos.org. When you're ready to submit your revision, log on to https://www.editorialmanager.com/pone/ and select the 'Submissions Needing Revision' folder to locate your manuscript file.

Please include the following items when submitting your revised manuscript:

- A rebuttal letter that responds to each point raised by the academic editor and reviewer(s). You should upload this letter as a separate file labeled 'Response to Reviewers'.
- A marked-up copy of your manuscript that highlights changes made to the original version. You should upload this as a separate file labeled 'Revised Manuscript with Track Changes'.
- An unmarked version of your revised paper without tracked changes. You should upload this as a separate file labeled 'Manuscript'.

If you would like to make changes to your financial disclosure, please include your updated statement in your cover letter. Guidelines for resubmitting your figure files are available below the reviewer comments at the end of this letter.

If applicable, we recommend that you deposit your laboratory protocols in protocols.io to enhance the reproducibility of your results. Protocols.io assigns your protocol its own identifier (DOI) so that it can be cited independently in the future. For instructions see: http://journals.plos.org/plosone/s/submission-guidelines#loc-laboratory-protocols

We look forward to receiving your revised manuscript.

Kind regards,

Christophe Sola, Pharm.D., Ph.D.

Academic Editor

PLOS ONE

Journal Requirements:

When submitting your revision, we need you to address these additional requirements.

1. Please ensure that your manuscript meets PLOS ONE's style requirements, including those for file naming. The PLOS ONE style templates can be found at

https://journals.plos.org/plosone/s/file?id=wjVg/PLOSOne_formatting_sample_main_body.pdf and

https://journals.plos.org/plosone/s/file?id=ba62/PLOSOne_formatting_sample_title_authors_affiliations.pdf

2.Please include your tables as part of your main manuscript and remove the individual files. Please note that supplementary tables (should remain/ be uploaded) as separate "supporting information" files.

3.In your Data Availability statement, you have not specified where the minimal data set underlying the results described in your manuscript can be found. PLOS defines a study's minimal data set as the underlying data used to reach the conclusions drawn in the manuscript and any additional data required to replicate the reported study findings in their entirety. All PLOS journals require that the minimal data set be made fully available. For more information about our data policy, please see http://journals.plos.org/plosone/s/data-availability.

Upon re-submitting your revised manuscript, please upload your study’s minimal underlying data set as either Supporting Information files or to a stable, public repository and include the relevant URLs, DOIs, or accession numbers within your revised cover letter. For a list of acceptable repositories, please see http://journals.plos.org/plosone/s/data-availability#loc-recommended-repositories. Any potentially identifying patient information must be fully anonymized.

Important: If there are ethical or legal restrictions to sharing your data publicly, please explain these restrictions in detail. Please see our guidelines for more information on what we consider unacceptable restrictions to publicly sharing data: http://journals.plos.org/plosone/s/data-availability#loc-unacceptable-data-access-restrictions. Note that it is not acceptable for the authors to be the sole named individuals responsible for ensuring data access.

We will update your Data Availability statement to reflect the information you provide in your cover letter.

4.PLOS ONE now requires that authors provide the original uncropped and unadjusted images underlying all blot or gel results reported in a submission’s figures or Supporting Information files. This policy and the journal’s other requirements for blot/gel reporting and figure preparation are described in detail at https://journals.plos.org/plosone/s/figures#loc-blot-and-gel-reporting-requirements and https://journals.plos.org/plosone/s/figures#loc-preparing-figures-from-image-files. When you submit your revised manuscript, please ensure that your figures adhere fully to these guidelines and provide the original underlying images for all blot or gel data reported in your submission. See the following link for instructions on providing the original image data: https://journals.plos.org/plosone/s/figures#loc-original-images-for-blots-and-gels.

In your cover letter, please note whether your blot/gel image data are in Supporting Information or posted at a public data repository, provide the repository URL if relevant, and provide specific details as to which raw blot/gel images, if any, are not available. Email us at plosone@plos.org if you have any questions.

5. PLOS requires an ORCID iD for the corresponding author in Editorial Manager on papers submitted after December 6th, 2016. Please ensure that you have an ORCID iD and that it is validated in Editorial Manager. To do this, go to ‘Update my Information’ (in the upper left-hand corner of the main menu), and click on the Fetch/Validate link next to the ORCID field. This will take you to the ORCID site and allow you to create a new iD or authenticate a pre-existing iD in Editorial Manager. Please see the following video for instructions on linking an ORCID iD to your Editorial Manager account: https://www.youtube.com/watch?v=_xcclfuvtxQ

[Note: HTML markup is below. Please do not edit.]

Reviewers' comments:

Reviewer's Responses to Questions

**Comments to the Author**

1. Is the manuscript technically sound, and do the data support the conclusions?

The manuscript must describe a technically sound piece of scientific research with data that supports the conclusions. Experiments must have been conducted rigorously, with appropriate controls, replication, and sample sizes. The conclusions must be drawn appropriately based on the data presented. 

Reviewer #1: Yes

**********

2. Has the statistical analysis been performed appropriately and rigorously? 

Reviewer #1: N/A

**********

3. Have the authors made all data underlying the findings in their manuscript fully available?

The PLOS Data policy requires authors to make all data underlying the findings described in their manuscript fully available without restriction, with rare exception (please refer to the Data Availability Statement in the manuscript PDF file). The data should be provided as part of the manuscript or its supporting information, or deposited to a public repository. For example, in addition to summary statistics, the data points behind means, medians and variance measures should be available. If there are restrictions on publicly sharing data—e.g. participant privacy or use of data from a third party—those must be specified.

Reviewer #1: Yes

**********

4. Is the manuscript presented in an intelligible fashion and written in standard English?

PLOS ONE does not copyedit accepted manuscripts, so the language in submitted articles must be clear, correct, and unambiguous. Any typographical or grammatical errors should be corrected at revision, so please note any specific errors here.

Reviewer #1: Yes

**********

5. Review Comments to the Author

Please use the space provided to explain your answers to the questions above. You may also include additional comments for the author, including concerns about dual publication, research ethics, or publication ethics. (Please upload your review as an attachment if it exceeds 20,000 characters)

Reviewer #1: Tuberculosis is a serious public health problem, especially for people living in poor and developing regions, and because of the ability of M. tuberculosis to infect virtually all organs of the human body, its diagnosis in cases of extra-pulmonary tuberculosis has played a challenge. In the present manuscript we are faced with a “challenge within a challenge” which is the diagnosis of tuberculous meningitis, a severe form of tuberculosis (paucibacillary) with a high rate (1 - 5%) of involvement, as shown by the authors. The manuscript's theme is of great importance for the control of tuberculosis, and the results in a way present guideline for a better management of cases of tuberculous meningitis, also using the detection of resistant isolates.

The manuscript is rather concise in their proposal and presents the results accurately, however is full of small mismatches, such as: a) MGIT 960 is presented in two ways: MGIT 960 and MGIT960; b) In the method section it is stated that the 2 mL of the CSF were divided into 1.5 mL for Xpert and 0.5 mL for MGIT, then it says that 2 mL were used for Xpert; c) To standardize the wording of units in relation to their values, or with space or without space, observe the recommendations of PLOS; d) Standardize the wording of the names of the drugs, or start with a capital letter or a small letter; e) Check if the parameters of the multiplex PCR for the rpoB, inhA and katG genes are correct; f) I recommend a careful review of the references, mainly related to how the species M. tuberculosis is being written; g) The highlighted isolates (GC3, GC13 and GC16) throughout the text and in the legends are spelled in different ways; h) The purpose of the presentation of Figure 2B is not clear, since the electropherograms do not correspond to the sequences presented for the M. tuberculosis H37Rv strain, and the clinical isolates GC6 and GC16; i) Figure 3 shows the scientific quality and accuracy of the results obtained in this study, however it does not contribute to a better understanding of the work, I recommend removing it; j) MIRU-VNTR is recognized as a fundamental tool for the molecular characterization of M. tuberculosis isolates and has been widely used for this purpose, therefore it is not necessary to include the table of primers used for PCR reactions, unless this work proposed some modifications in these sequences; k) Although it is not the objective of this work, I believe it is important to include in the discussion information that discards epidemiological links of the patients involved in this study; l) In this manuscript the authors refer to: “confirmation of isolates of MBTC” and “identify of MBTC” however this acronym is not defined throughout the text, if this acronym refers to the M. tuberculosis Complex, consider the adequacy as the publication by Riojas et al., Int J Syst Evol Microbiol 2018; 68: 324–332.

Reviewer #2: accept as is.

**********

6. PLOS authors have the option to publish the peer review history of their article (what does this mean?). If published, this will include your full peer review and any attached files.

If you choose “no”, your identity will remain anonymous but your review may still be made public.

**Do you want your identity to be public for this peer review?** For information about this choice, including consent withdrawal, please see our Privacy Policy.

Reviewer #1: No

[NOTE: If reviewer comments were submitted as an attachment file, they will be attached to this email and accessible via the submission site. Please log into your account, locate the manuscript record, and check for the action link "View Attachments". If this link does not appear, there are no attachment files.]

While revising your submission, please upload your figure files to the Preflight Analysis and Conversion Engine (PACE) digital diagnostic tool, https://pacev2.apexcovantage.com/. PACE helps ensure that figures meet PLOS requirements. To use PACE, you must first register as a user. Registration is free. Then, login and navigate to the UPLOAD tab, where you will find detailed instructions on how to use the tool. If you encounter any issues or have any questions when using PACE, please email PLOS at figures@plos.org. Please note that Supporting Information files do not need this step.

# Author comment: Author response to Decision Letter 0

**DOI:** 10.1371/journal.pone.0240257.r002

4 Sep 2020

Reviewer #1: 

Thank you so much for reviewing our manuscript and your suggestions are very helpful. As per the reviewer’s suggestions, we revised the manuscript accordingly and highlighted the corrections.

a) MGIT 960 is presented in two ways: MGIT 960 and MGIT960

We presented MGIT 960 as MGIT 960 throughout the manuscript 

b) In the method section it is stated that the 2 mL of the CSF were divided into 1.5 mL for Xpert and 0.5 mL for MGIT, then it says that 2 mL were used for Xpert

We have written this protocol elaborately in GeneXpert methodology section. The 1.5 ml of CSF samples diluted with GeneXpert MTB/RIF sample reagent (1.5 ml) was vortexed for 30 seconds and incubated at room temperature for 10 minutes. The CSF-reagent mixture was vortexed again for 30 seconds and then incubated for 5 minutes. The 2 ml of CSF-reagent mixture was then transferred into the GeneXpert MTB/RIF cartridge and loaded into the GeneXpert equipment. Kindly find the revised sentence in the page no 4 

c) To standardize the wording of units in relation to their values, or with space or without space, observe the recommendations of PLOS

As per the guidelines of PLOS, we provided the space to units in relation to their values. Kindly find the corrections in the page no’s 4, 5, 6 and 9

d) Standardize the wording of the names of the drugs, or start with a capital letter or a small letter 

We started the names of all the drugs with a small letter. SIRE was written as streptomycin, isoniazid, rifampicin and ethambutol. Kindly find the changes in the page no’s 4 and 6 

e) Check if the parameters of the multiplex PCR for the rpoB, inhA and katG genes are correct

As per the reviewer’s suggestion, we have checked the parameters of the multiplex PCR for the rpoB, inhA and katG genes and corrected appropriately. Please find the changes in page 6

f) I recommend a careful review of the references, mainly related to how the species M. tuberculosis is being written; 

As per the reviewer’s suggestion, we revised the reference very carefully and italicised Mycobacterium tuberculosis throughout the reference section. 

g) The highlighted isolates (GC3, GC13 and GC16) throughout the text and in the legends are spelled in different ways

Thank you so much for this comment. We corrected the isolates no’s as GC6, GC13 and GC16. Kindly find the changes in the supporting information in (S1-S3 Figs in S1File).

h) The purpose of the presentation of Figure 2B is not clear, since the electropherograms do not correspond to the sequences presented for the M. tuberculosis H37Rv strain, and the clinical isolates GC6 and GC16

To analyse the mutations associated with RIF resistance, the rifampicin resistance-determining region (RRDR) was sequenced after amplification of rpoB gene by conventional PCR by using the primers, rpoBF (5’CAGACGTTGATCAACATCCG3’) and rpoBR (5’TACGGCGTTTCGATGAAC3’) to generate 305 bp that contains 81bp hot-spot RRDR (Page No 6). The rpoB gene was sequenced using reverse primer and the electropherogram of RRDR sequence was presented as 3’ to 5’ (S2 Fig in S1 File). During mutation analysis, we have converted the sequence into 5’ to 3’ (S3 Fig in S1 File). We revised the figures accordingly. Kindly find the changes in the S1 File. We compared the rpoB sequence of GC6 and GC16 strains with H37Rv rpoB (KY702773.1)

i) Figure 3 shows the scientific quality and accuracy of the results obtained in this study, however it does not contribute to a better understanding of the work, I recommend removing it

As per the reviewer’s suggestion, we removed the figure 3 from the manuscript 

j) MIRU-VNTR is recognized as a fundamental tool for the molecular characterization of M. tuberculosis isolates and has been widely used for this purpose, therefore it is not necessary to include the table of primers used for PCR reactions, unless this work proposed some modifications in these sequences

As per the reviewer’s suggestion, we removed the Primers list from the manuscript. However, we included the references in the MIRU-VNTR methodology section. 

k) Although it is not the objective of this work, I believe it is important to include in the discussion information that discards epidemiological links of the patients involved in this study

 Thank you so much for your comments. We aimed this study to validate the diagnostic performance of GeneXpert to diagnose TBM by using MGIT 960 as the gold standard, in addition to identify the genotypes and drug susceptibility profiling of circulating Mtb strains among TBM patients in south India. The discordant results between GeneXpert, MGIT 960 and Line probe assay with respect to rifampicin resistance was highlighted. Hence, we didn’t discuss the epidemiological link of the TBM patients. In the present study, we suggest that despite the presence of diverse genotypes of Mtb strains in south India, the EAI family is the most predominant Mtb genotype linked to drug resistant TB among south Indian TBM patients. We consider that the EAI family is driving drug resistant TB in south India and assume that its role in association with multi drug resistant TB will increase in the future. However, epidemiological surveillance of these cases in future may find new patients from their epidemiological links, predict risk factors and develop strategies to control and for the management of drug resistant TB in south India. 

l) In this manuscript the authors refer to: “confirmation of isolates of MBTC” and “identify of MBTC” however this acronym is not defined throughout the text, if this acronym refers to the M. tuberculosis Complex, consider the adequacy as the publication by Riojas et al., Int J Syst Evol Microbiol 2018; 68: 324–332.

The acronym of Mycobacterium tuberculosis complex is MTBC. We made the changes in the manuscript and highlighted in the page 5. 

Reviewer #2: accept as is.

Thank you so much for your valuable time and review

Attachment Submitted filename: Response to reivewers 04-09-2020.docx Click here for additional data file.

# Editor report: Decision Letter 1

**DOI:** 10.1371/journal.pone.0240257.r003

**Authors:** Christophe Sola

23 Sep 2020

Molecular diagnosis, genetic diversity and drug sensitivity patterns of Mycobacterium tuberculosis strains isolated from tuberculous meningitis patients at a tertiary care hospital in South India

PONE-D-20-08897R1

Dear Dr. Joseph,

We’re pleased to inform you that your manuscript has been judged scientifically suitable for publication and will be formally accepted for publication once it meets all outstanding technical requirements.

Within one week, you’ll receive an e-mail detailing the required amendments. When these have been addressed, you’ll receive a formal acceptance letter and your manuscript will be scheduled for publication.

An invoice for payment will follow shortly after the formal acceptance. To ensure an efficient process, please log into Editorial Manager at http://www.editorialmanager.com/pone/, click the 'Update My Information' link at the top of the page, and double check that your user information is up-to-date. If you have any billing related questions, please contact our Author Billing department directly at authorbilling@plos.org.

If your institution or institutions have a press office, please notify them about your upcoming paper to help maximize its impact. If they’ll be preparing press materials, please inform our press team as soon as possible -- no later than 48 hours after receiving the formal acceptance. Your manuscript will remain under strict press embargo until 2 pm Eastern Time on the date of publication. For more information, please contact onepress@plos.org.

Kind regards,

Christophe Sola, Pharm.D., Ph.D.

Academic Editor

PLOS ONE

Additional Editor Comments (optional):

The minor changes were adequately made by the authors.

please take care to correct english spelling of some words: pathogenesis not pathogeneses

correct some remaining shells

et al. : italicized

use brand names only once, (first time only) with city and country of origin : genoLyse (Hain LifeSciences, Nehren, Germany) as an example, same for all commercial names

Eppendorf not eppendorf

verify carefully all sentences (grammar, orthograph)

Reviewers' comments: no more comment, to be accepted as is

# Editor report: Acceptance letter

**DOI:** 10.1371/journal.pone.0240257.r004

**Authors:** Christophe Sola

25 Sep 2020

PONE-D-20-08897R1 

Molecular diagnosis, genetic diversity and drug sensitivity patterns of Mycobacterium tuberculosis strains isolated from tuberculous meningitis patients at a tertiary care hospital in South India 

Dear Dr. Joseph:

I'm pleased to inform you that your manuscript has been deemed suitable for publication in PLOS ONE. Congratulations! Your manuscript is now with our production department. 

If your institution or institutions have a press office, please let them know about your upcoming paper now to help maximize its impact. If they'll be preparing press materials, please inform our press team within the next 48 hours. Your manuscript will remain under strict press embargo until 2 pm Eastern Time on the date of publication. For more information please contact onepress@plos.org.

If we can help with anything else, please email us at plosone@plos.org. 

Thank you for submitting your work to PLOS ONE and supporting open access. 

Kind regards, 

PLOS ONE Editorial Office Staff

on behalf of

Pr. Christophe Sola 

Academic Editor

PLOS ONE
